# Oxidative and Anti-Oxidative Stress Markers in Chronic Glaucoma: A Systematic Review and Meta-Analysis

**DOI:** 10.1371/journal.pone.0166915

**Published:** 2016-12-01

**Authors:** Cédric Benoist d’Azy, Bruno Pereira, Frédéric Chiambaretta, Frédéric Dutheil

**Affiliations:** 1 University Hospital of Clermont-Ferrand (CHU), Ophthalmology, Clermont-Ferrand, France; 2 University Hospital of Clermont-Ferrand (CHU), Preventive and Occupational Medicine, Clermont-Ferrand, France; 3 University Hospital of Clermont-Ferrand (CHU), Clinical Research Direction, Clermont-Ferrand, France; 4 CNRS Physiological and Psychosocial Stress, LAPSCO, University Clermont Auvergne, Clermont-Ferrand, France; 5 Australian Catholic University, Faculty of Health, School of Exercise Science, Melbourne, Australia; 6 University Clermont Auvergne, Laboratory of Metabolic Adaptations to Exercise in Physiological and Pathological conditions EA3533, Clermont-Ferrand, France; 7 Research Centre in Human Nutrition (CRNH) Auvergne, Clermont-Ferrand, France; Kermanshah University of Medical Sciences, ISLAMIC REPUBLIC OF IRAN

## Abstract

Chronic glaucoma is a multifactorial disease among which oxidative stress may play a major pathophysiological role. We conducted a systematic review and meta-analysis to evaluate the levels of oxidative and antioxidative stress markers in chronic glaucoma compared with a control group. The PubMed, Cochrane Library, Embase and Science Direct databases were searched for studies reporting oxidative and antioxidative stress markers in chronic glaucoma and in healthy controls using the following keywords: “oxidative stress” or “oxidant stress” or “nitrative stress” or “oxidative damage” or “nitrative damage” or “antioxidative stress” or “antioxidant stress” or “antinitrative stress” and “glaucoma”. We stratified our meta-analysis on the type of biomarkers, the type of glaucoma, and the origin of the sample (serum or aqueous humor). We included 22 case-control studies with a total of 2913 patients: 1614 with glaucoma and 1319 healthy controls. We included 12 studies in the meta-analysis on oxidative stress markers and 19 on antioxidative stress markers. We demonstrated an overall increase in oxidative stress markers in glaucoma (effect size = 1.64; 95%CI 1.20–2.09), ranging from an effect size of 1.29 in serum (95%CI 0.84–1.74) to 2.62 in aqueous humor (95%CI 1.60–3.65). Despite a decrease in antioxidative stress marker in serum (effect size = –0.41; 95%CI –0.72 to –0.11), some increased in aqueous humor (superoxide dismutase, effect size = 3.53; 95%CI 1.20–5.85 and glutathione peroxidase, effect size = 6.60; 95%CI 3.88–9.31). The differences in the serum levels of oxidative stress markers between glaucoma patients and controls were significantly higher in primary open angle glaucoma vs primary angle closed glaucoma (effect size = 12.7; 95%CI 8.78–16.6, P < 0.001), and higher in pseudo-exfoliative glaucoma vs primary angle closed glaucoma (effect size = 12.2; 95%CI 8.96–15.5, P < 0.001). In conclusion, oxidative stress increased in glaucoma, both in serum and aqueous humor. Malonyldialdehyde seemed the best biomarkers of oxidative stress in serum. The increase of some antioxidant markers could be a protective response of the eye against oxidative stress.

## Introduction

Chronic glaucoma is one of the most frequently established diseases in ophthalmology and represents a growing public health concern, with consequences that lead to blindness [1,2]. This pathology causes significant impact on visual function that may affect the quality of life and work productivity [3,4]. Due to its long time asymptomatic evolution, this disease remains under-diagnosed and the irreversible loss of optic nerve fibers leads to important visual field loss [5]. Chronic glaucoma is a multifactorial disease implicating divers factors such as intraocular pressure, familial history, myopia, corneal thickness and ethnicity [6,7,8,9,10]. Extensive research over the last three decades has demonstrated that oxidative stress can cause peroxidation of nucleic acids, bases, lipids, proteins and carbohydrates, thus resulting in their damage. At the ocular level, oxidative stress has been postulated to promote chronic glaucoma [11]. Conversely, antioxidants could protect against glaucoma [12].

However, oxidative stress is not assessed in daily clinical practice [13]. Reactive oxygen species are so reactive and so short-lived that they are difficult to measure directly [13]. There are numerous markers of oxidative stress, and several methods of measurement. In glaucoma, oxidative stress can be assessed both in the serum or the aqueous humor. Although the evaluation of different markers of oxidative and antioxidative stress in glaucoma has been assessed in several studies and discussed in several reviews [14,15,16,17], the results are under debate. We hypothesized that glaucoma patients would exhibit an increased level of oxidative stress and a decreased level of antioxidative markers.

Thus, we aimed to conduct a systematic review and meta-analysis to summarize all studies reporting the level of oxidative and antioxidative markers in aqueous humor or serum samples of glaucoma patients compared to controls.

## Methods

### Literature search

**Fig 1:**
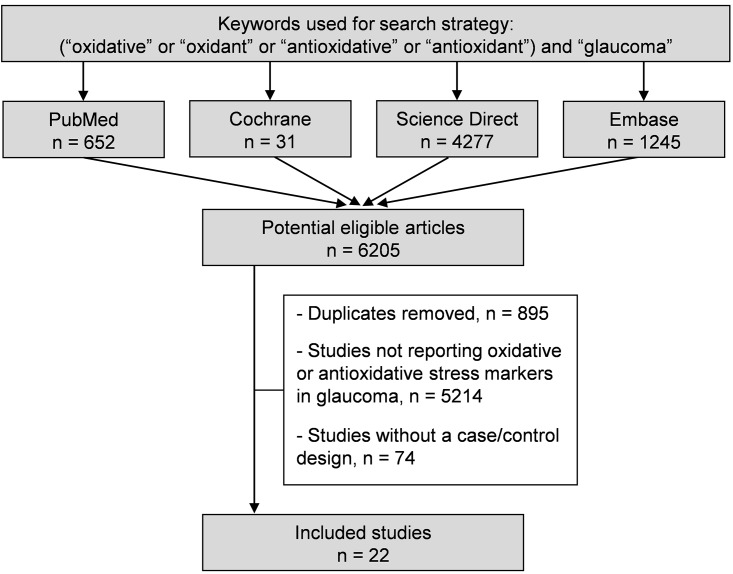
Search strategy.

We reviewed all studies measuring oxidative or antioxidative stress markers in chronic glaucoma patients and controls. Animal studies were excluded. The PubMed, Cochrane Library, Science Direct and Embase databases were searched on May 10^th^ 2016, with the following keywords: “oxidative stress” or “oxidant stress” or “nitrative stress” or “oxidative damage” or “nitrative damage” or “antioxidative stress” or “antioxidant stress” or “antinitrative stress” and “glaucoma”. The search was not limited to specific years. No minimal sample size and no language restrictions were applied. To be included, articles needed to be case-control studies describing our primary outcome variable, which was the measurement of oxidative or antioxidative stress markers in glaucoma patients and healthy controls. We imposed no limitation on the regional origin or the nature of the control group. Studies needed to be primary research. In addition, reference lists of all publications meeting the inclusion criteria were manually searched to identify any further studies that were not found with the electronic search. Ancestry searches were also completed on previous reviews to locate other potential eligible primary studies. The search strategy is presented in Fig 1. One author (CBDA) conducted all literature searches and collated the abstracts. Two authors (CBDA and FD) separately reviewed the abstracts and based on the selection criteria, decided the suitability of the articles for inclusion. A third author (BP) was asked to review the articles where consensus on suitability was debated. All authors then reviewed the eligible articles.

### Quality of assessment

Although not designed for quantifying the integrity of studies [18], the “STrengthening the Reporting of OBservational studies in Epidemiology” (STROBE) criteria were used to check the quality of the reporting [19]. The STROBE Statement consists of a checklist of 22 items, which relate to the title, abstract, introduction, methods, results and discussion sections of articles. Eighteen items are common to cohort studies, case control studies and cross-sectional studies and four are specific to each of the three study designs. Among the 22 items, six are split into several sub-items. One point was attributed per item or sub-item when the study fulfilled the criteria. The maximum score achievable was 32, then converted into percentage.

### Statistical considerations

Statistical analysis was conducted using Comprehensive Meta-analysis software (version 2, Biostat Corporation) [20,21,22,23] and Stata software (version 13, StataCorp, College Station, US). Baseline characteristics were summarized for each study sample and reported as mean (standard-deviation) and number (%) for continuous and categorical variables respectively. Heterogeneity in the study results was evaluated by examining forest plots, confidence intervals (CI) and using formal tests for homogeneity based on the I² statistic, which is the most common metric for measuring the magnitude of between-study heterogeneity and is easily interpretable. I² values range between 0% and 100% and are typically considered low for <25%, modest for 25–50%, and high for >50%. This statistical method generally assumes heterogeneity when the p-value of the I² test is <0.05. For example, a significant heterogeneity may be due to the variability between the characteristics of the studies such as those of the participants (age, sex, etc), the type of biomarkers, the type of glaucoma, or the origin of the sample (aqueous humor or serum). Random effects meta-analyses (DerSimonian and Laird approach) were conducted when data could be pooled [24]. P values less than 0.05 were considered statistically significant.

We conducted meta-analysis on the levels of oxidative and antioxidative stress markers in chronic glaucoma patients and healthy controls. We stratified these meta-analyses on the type of biomarkers, the type of glaucoma, and the sample origin (aqueous humor or serum). We described our results by calculating the effect size (ES, standardized mean differences—SMD) of the oxidative and antioxidative stress markers for each dependent variable [24]. An ES is defined as a unitless measure of the levels of the stress markers centered at zero if the stress marker levels in chronic glaucoma patients are not different from those in healthy controls. A positive ES denoted improved performance. A scale for ES has been suggested with 0.8 reflecting a large effect, 0.5 a moderate effect, and 0.2 a small effect [25].

For rigor, funnel plots of these meta-analyses were used to search for potential publication bias. In order to verify the strength of the results, further meta-analyses were then conducted excluding studies that were not evenly distributed around the base of the funnel [26].

When possible (sufficient sample size), meta-regressions were proposed to study the relationship between the levels of oxidative and antioxidative stress markers and clinically relevant parameters such as the participants gender, age, the type of biomarkers, the type of glaucoma, and the sample origin (aqueous humor or serum). Results were expressed as regression coefficients and 95%CI.

## Results

An initial search produced a possible 9,058 articles (Fig 1). Removal of duplicates and use of the selection criteria reduced the number of articles reporting the evaluation of oxidative or antioxidative markers in aqueous humor or blood to 22 articles [27–48]. All articles were written in English.

### Quality of articles

The assessment of the quality of the 22 studies that were included was performed using the STROBE criteria, with the results varying from 68.8 [45] to 90.6% [32,46], with a mean score of 80.3±6.3. Overall, the studies performed best in the methods section and worst in the discussion section. All studies except one mentioned ethical approval [43].

### Method of sampling for markers analysis

Levels of oxidant or antioxidant markers were evaluated in serum in 12 studies [27,28,29,31,32,34,35,39,40,43,45,48], in aqueous humor in six studies [30,36,37,38,44,47], or both in three studies [33,41,42]. One study measured the markers in blood hemolysate [46]. Blood samples were collected in ethylenediaminetetraacetic acid tubes. The tubes were centrifuged and the plasma layer was separated and stored until analysis. At the beginning of the surgery, 0.1–0.2 ml of aqueous humor samples were obtained from each patient through a paracentesis using a 27 gauge needle. Patients were those requiring either glaucoma surgery (glaucoma group) or cataract surgery (control group) in six studies [30,36,37,42,44,47] or only before cataract surgery (glaucoma and control group) in three studies [33,38,41]. Aqueous humor samples were immediately frozen at -80°C until processing for the subsequent biochemistry techniques.

### Inclusion criteria for glaucoma patients

The markers were measured in different types of glaucoma: only in primary open angle glaucoma (POAG) [29,30,36,37,39,41,43,44,45,46,48], only in primary angle closure glaucoma (PACG) [27,31], only in pseudoexfoliation glaucoma (PEG) [28,32,33], in both POAG and PACG [38], in both POAG and PEG [35,42,47], in both POAG, PACG and PEG [40], or it was not specified [34]. Inclusion criteria for glaucoma patients were similar for most studies [27–33,35–39,41,43,46,47]. Patients had to have a vertical cup/disc ratio of 0.5 or higher and a typical glaucomatous defect at the visual field (standard automatized visual field analyser). Some specificities were required for each type of glaucoma, such as an open angle for the gonioscopy for POAG [29,30,36,37,38,39,41,43,46,48], a close angle for PACG [27,31,38], or clinical evidence of exfoliative material on the pupil margin or anterior lens surface for PEG [28,32,33,35,47]. Some studies required an elevation of the intraocular pressure for glaucoma patients [27,28,32,33,35,37,38,43,48]. Criteria for glaucoma were not defined in five studies [34,40,42,44,45].

### Exclusion criteria

Eight studies excluded patients with hypertension, dyslipemia, endocrinopathy, autoimmune disease, liver or kidney or heart failure, malignant tumor, or metabolic diseases such as diabetes [31,36,37,39,40,41,43,44,46,47,48]. Seven studies excluded smokers or patients with a history of smoking [32,37,38,40,41,43,44]. Six studies excluded medications with non-steroidal anti-inflammatory agent [32,37,38,39,43,44]. Eight studies excluded patients on special diets with antioxidant vitamins such as vitamin C and E [32,37,38,41,43,44,47,48]. Patients with previous intraocular surgery were excluded in eight studies [31,36,37,39,45,46,47,48].

### Population

#### Sample size

Population sizes ranged from 20 [30] to 691 [40]. We included 2,913 patients in total: 1,614 with glaucoma and 1,319 healthy controls. The proportion of patients with glaucoma varied between 40.0 [43] to 83.8% [34].

#### Gender

The proportion of men varied between 33.8 [34] to 68.5% [28] in the glaucoma group and from 25.0 [43] to 68.5% [28] in the control group. One study did not specify the proportion of men [38].

#### Age

The mean age in the glaucoma group was 59.4±7.5 years (ranging from 51.0±14.2 [34] to 74.9±7.5 [30]) and 63.2±6.2 years in the control group (ranging from 44.9±10.8 [34] to 75.3±9.1 [41]).

#### Glaucoma characterization

Ten studies reported intraocular pressure in the glaucoma group [27,29,30,31,36,37,38,39,40,42,46,48], ranging from 13.4±1.6 [39] to 30.45±4.01 mmHg [38]. Five studies reported intraocular pressure in controls, ranging from 14.0±6.0 [36,47] to 18.6±5.3 mmHg [37]. The cup/disc ratio was reported in glaucoma group in 6 studies [27,29,36,37,40,42,46,48], ranging from 0.67±0.22 [29] to 0.90±0.10 [36]. Only one study reported the cup/disc ratio in controls [37]. Three studies reported the number of medications for treating glaucoma [27,29,30], ranging from 1.9±1.6 [27] to 3.0±0.0 [30] medications.

### Outcome and aim of the studies

The principal aim of all the studies included was to evaluate the level of antioxidant or oxidant markers in glaucoma patients.

### Study designs

All studies described a monocentric, prospective, case-controlled study comparing levels of antioxidative or oxidative stress markers in glaucoma patients with aged and sex-matched controls. When aqueous humor sampling was needed, controls were recruited among individuals scheduled for cataract surgery.

### Oxidant markers and condition of analysis

Total oxidative stress was reported in four studies. Serum total oxidative stress levels were measured according to the method described by Erel using a commercially available kit (Relassay, Turkey) [32,33,35] or by measuring the organic hydroperoxides that are related to the free radicals from which they are formed [48].

Malonyldialdehyde was reported in eight studies. In serum, malonyldialdehyde levels were measured with the thiobarbituric acid reaction as described by Yagi [31,34,35,43], or by high performance liquid chromatography [41]. In aqueous humor, malonyldialdehyde levels were determined using the thiobarbituric acid reaction [37,41,44]. One study assessed malonyldialdehyde levels in blood hemolysate using the method described by Ohkawa et al [46].

Advanced oxidation protein product was reported in two studies [31,34], and measured with the use of the AU 2700 autoanalyser (Olympus Diagnostics Inc. Melville, NY)[34] or quantified as described by Witko-Sarsat *et al* [31]

8-hydroxydeoxyguanosin (8-OHdG) was reported in two studies, and measured in serum and aqueous humor using an enzyme-linked immunosorbent assay (8-OHdG check; Japan Institute for the Control of Aging, Shizuko, Japan) [31,42].

Protein carbonyl was reported in two studies and was measured using the spectrophotometric assay described by Reznick and Packe [49] or Levine *et al* [35].

Nitric oxide synthase was reported in two studies and was measured in serum using the Griess reaction [35], and using Clontech Ab Microarray 500 (Clontech, CA, USA) and Explorer Antibody Microarray (Full Moon BioSystems, Inc., Sunnyvale,CA, USA) in aqueous humor [30].

Other markers were reported in only one study. *4-hydroxynonenal (4-HNE)* was measured using an enzyme-linked immunosorbent assay method [31]. *Ischemied modified albumin* was analyzed using the rapid and colorimetric method described by Bar-Or *et al* [31]. *Conjugated diene* was determined using the method of Ward *et al* [31] *Myeloperoxidase* was measured from the reduction of o-dianozidine by spectrophotometer [43]. *Glutamine synthase* was analyzed using Clontech Ab Microarray 500 (Clontech, CA, USA) and Explorer Antibody Microarray (Full Moon BioSystems, Inc., Sunnyvale,CA, USA).[30]

### Antioxidant markers and analysis conditions

Total antioxidant status was measured in 14 studies using a Colorimetric-based assay available from Randox (Randox Laboratories Ltd, UK) [27,28,29,34,39,40,42,48], an automated colorimetric measurement method developed by Erel [32,33,35], the oxygen-radical absorbance capacity [41], or chemiluminescence [36,47].

Superoxide dismutase was reported in ten studies. In serum, it was measured using the method described by Sun *et al* [35,45], the procedure of Misra and Ribovich [39], the Oyanagui method [46], or using the Roche COBAS MIRA Plus Chemistry Analyzer (Roche Diagnostics Ltd. W Sussex, UK) [34]. In aqueous humor, it was determined spectrophotometrically [30,36,37,38,47].

Glutathione peroxidase was reported in nine studies. In serum, it was measured using a cellular glutathione peroxidase kit (Cayman Chemical Ann Arbor, MI) modified for the Roche COBAS MIRA Plus analyzer (Roche Diagnostics Ltd. W Sussex, UK) [34], using a spectrophotometric procedure by Little and O'Brian (1968) [39] or by Sedlak and Lindsay [45], or as described by Paglia *et al* [46]. In aqueous humor, glutathione peroxidase was determined by reduced nicotinamide adenine dinucleotide phosphate oxidation at 340 nm [30,36,37,38,47].

Catalase was reported in eight studies. In serum, it was evaluated by the spectrophotometric procedure of Beers and Sizer (1952) [39,45] and the method defined by Beutler [43] or Aebi [46]. In aqueous humor, catalase activity was determined by measuring the decrease in H_2_O_2_ absorbance at 240 nm in a reaction mix consisting of 100 mmol/L phosphate buffer (pH 7.2) and 10 mmol/l hydrogen peroxide [36,37,38,47].

Paraoxonase and arylesterase were measured in two studies using methods by Kirbas [33] or commercially available kits (Relassay, Turkey) [32].

Vitamin C was measured in two studies in aqueous humor with a spectrophotometer according to the method described by Kyaw [38,47], and in one study in serum using the dinitrophenylhydrazine method developed by Roe and Kuether [45].

Other markers were reported in only one study. In serum, *vitamins A* and *E* were measured using high performance liquid chromatography [34]. In aqueous humor, vitamin E was estimated spectrophotometrically according to the method described by Desai and Kyaw [38]. *Transferrin* was measured using a Beckman kit [34]. The level of *glutathione S transferase* was determined by the method developed by Habig *et al* [45].

### Meta-analyses of oxidative stress in glaucoma

#### Overall

Twelve studies were included in the overall meta-analysis of the levels of oxidative stress markers in the serum and aqueous humor of glaucoma patients compared with controls [30–35,37,41–44,46]. The effect size of oxidative stress markers in the glaucoma group was 1.64 (95%CI 1.20–2.09, P < 0.001) with an important heterogeneity (I^2^ = 94.6%, P < 0.001) (S1 Fig).

#### Stratification of oxidative stress markers in serum

**Fig 2:**
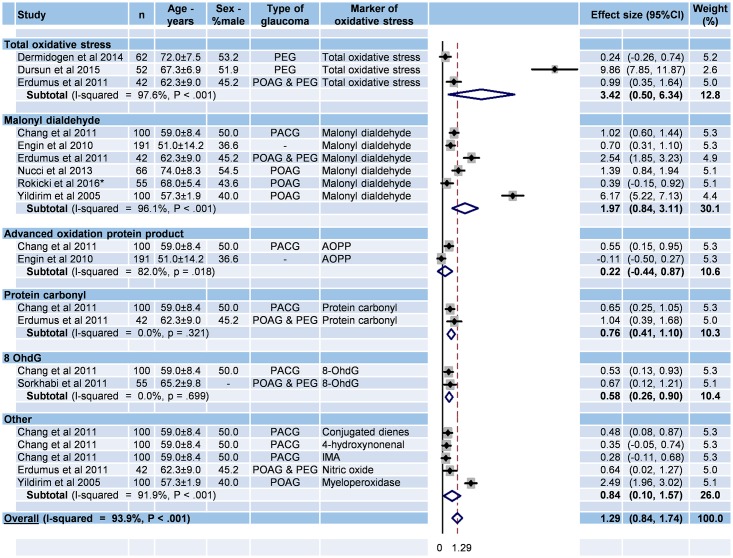
Meta-analysis of oxidative stress markers from serum in glaucoma. 95%CI: 95% confidence intervals; -: Unknown; PACG: primary angle closure glaucoma; PEG: pseudoexfoliation glaucoma; POAG: primary open angle glaucoma; 8 OhdG: 8-hydroxydeoxyguanosin; AOPP: Advanced oxidation protein product; IMA: Ischemied modified albumin.

Nine studies were included (Fig 2) [31,32,33,34,35,41,42,43,46]. The overall effect size of serum oxidative stress markers in glaucoma patients compared with controls was 1.29 (95%CI 0.84–1.74, P < 0.001; I^2^ = 93.9%, P < 0.001). More specifically, the effect sizes for total oxidative stress and malonyldialdehyde in glaucoma were 3.42 (95%CI 0.50–6.34, P = 0.022; I^2^ = 97.6%, P < 0.001) and 1.97 (95%CI 0.84–3.11, P = 0.001; I^2^ = 96.3%, P < 0.001), respectively. Other effect sizes were not significant.

#### Stratification of oxidative stress markers in aqueous humor

**Fig 3:**
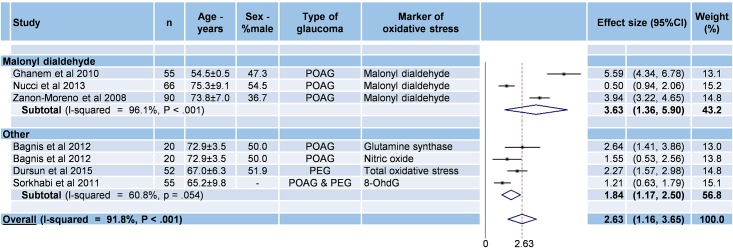
Meta-analysis of oxidative stress markers from aqueous humor in glaucoma. 95%CI: 95% confidence intervals; -: Unknown; PACG: primary angle closure glaucoma; PEG: pseudoexfoliation glaucoma; POAG: primary open angle glaucoma; 8 OhdG: 8-hydroxydeoxyguanosin.

Six studies were included (Fig 3) [30,33,37,41,42,44]. The overall effect size in aqueous humor in glaucoma patients compared with controls was 2.62 (95%CI 1.60–3.65, P < 0.001; I^2^ = 91.8%, P < 0.001), with an effect size for malonyldialdehyde of 3.63 (95%CI 1.36–5.90, P = 0.002; I^2^ = 96.1%, P < 0.001).

Funnel plots of previous meta-analyses analyzing for potential publication bias are presented in S2 Fig. For serum oxidative stress, meta-analysis was reperformed after the exclusion of studies that were not evenly distributed around the base of the funnel and which showed similar results (S3 Fig).

### Meta-analysis of antioxidative stress in glaucoma

#### Overall

Nineteen studies were included in the overall meta-analysis of the levels of antioxidative stress markers in the serum and aqueous humor of glaucoma patients compared with controls [27,28,29,30,32,33,34,35,36,37,38,39,40,41,42,43,44,46,47]. The effect size of antioxidative stress markers in the glaucoma group was –0.35 (95%CI –0.74 to 0.05, P = 0.089; I^2^ = 97.2%, P < 0.001) (S4 Fig).

#### Stratification of antioxidative markers in serum

**Fig 4:**
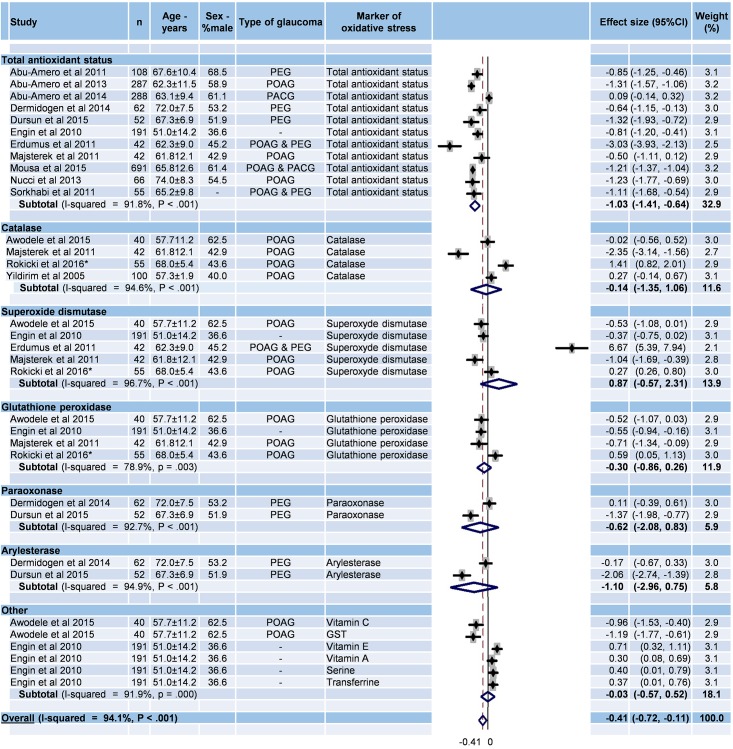
Meta-analysis of antioxidative stress markers from serum in glaucoma. 95%CI: 95% confidence intervals; -: Unknown; PACG: primary angle closure glaucoma; PEG: pseudoexfoliation glaucoma; POAG: primary open angle glaucoma; GST: Glutathione S transferase.

Fourteen studies were included (Fig 4) [27,28,29,32,33,34,35,39,40,41,42,43,45,46]. The overall effect size of serum antioxidative stress markers in glaucoma patients compared with controls was –0.41 (95%CI –0.72 to –0.11, P = 0.008; I^2^ = 94.1%, P < 0.001), with an effect size for total antioxidative status of –1.03 (95%CI –1.41 to –0.64, P < 0.001; I^2^ = 91.8%, P < 0.001). Other effect sizes were not significant.

#### Stratification of antioxidative markers in aqueous humor

**Fig 5:**
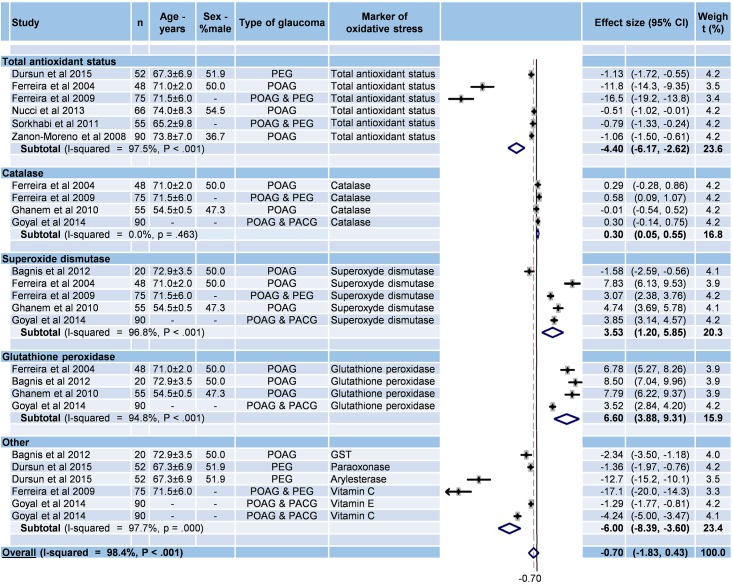
Meta-analysis of antioxidative stress markers from aqueous humor in glaucoma. 95%CI: 95% confidence intervals; -: Unknown; PACG: primary angle closure glaucoma; PEG: pseudoexfoliation glaucoma; POAG: primary open angle glaucoma; GST: Glutathione S transferase.

Nine studies were included (Fig 5) [30,33,36,37,38,41,42,44,47]. The overall effect size of serum oxidative stress markers in glaucoma patients compared with controls was non-significant (effect size = –0.70; 95%CI –1.83 to 0.43, P = 0.227; I^2^ = 98.4%, P < 0.001). However, the effect sizes were significant for total antioxidative status (effect size = –4.40; 95%CI –6.17 to –2.62, P < 0.001; I^2^ = 97.5%, P < 0.001), superoxide dismutase (effect size = 3.53; 95%CI 1.20–5.85, P 0.003; I^2^ = 96.8%, P < 0.001), and glutathione peroxydase (effect size = 6.60; 95%CI 3.88–9.31, P < 0.001; I^2^ = 94.8%, P < 0.001). The effect size of catalase was not significant (effect size = 0.30; 95%CI 0.05–0.55, p = 0.018, I^2^ = 0.0%, P = 0.463).

Funnel plots of previous meta-analyses analyzing for potential publication bias are presented in S5 Fig. For antioxidative stress in both serum and aqueous humor, meta-analyses were reperformed after the exclusion of studies that were not evenly distributed around the base of the funnel and which showed similar results (S6 and S7 Figs).

### Meta-analysis of oxidative markers for each type of glaucoma

**Fig 6:**
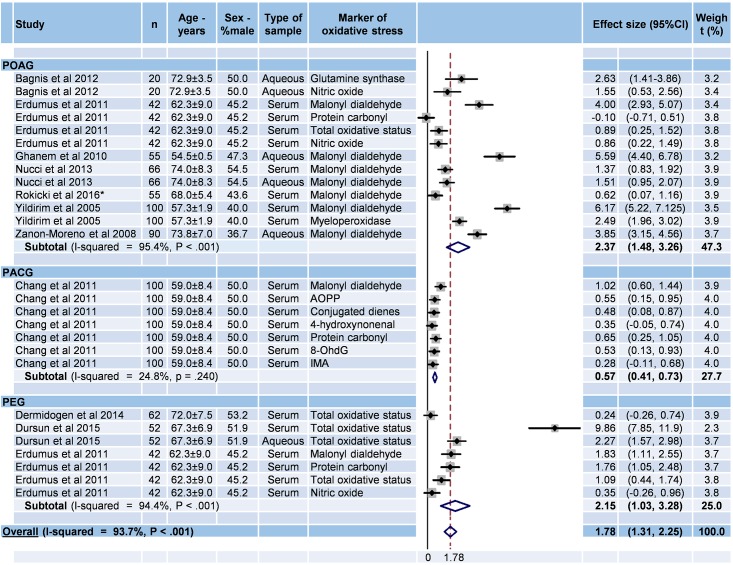
Meta-analysis of oxidative stress markers in glaucoma according to the type of glaucoma. 95%CI: 95% confidence intervals; -: Unknown; PACG: primary angle closure glaucoma; PEG: pseudoexfoliation glaucoma; POAG: primary open angle glaucoma; 8 OhdG: 8-hydroxydeoxyguanosin; AOPP: Advanced oxidation protein product; IMA: Ischemied modified albumin.

The 2 studies which did not specify the type of glaucoma were excluded.[34,42] Effect sizes were significant for the three types of glaucoma: 2.36 for POAG (95%CI 1.45–3.27, P < 0.001; I^2^ = 96.8%, P < 0.01), 0.55 for PACG (95%CI 0.37–0.72, P < 0.001; I^2^ = 94.8%, P < 0.01), and 2.15 for PEG (95%CI 1.03–3.28, P < 0.001; I^2^ = 97.7%, P < 0.01) (Fig 6).

### Metaregressions

#### Sex

Male glaucoma patients exhibited greater levels of oxidative stress markers in serum (effect size = 0.94; 95%CI 0.59–1.28, P < 0.001) (Table 1). Levels of oxidative stress markers in aqueous humor and antioxidative stress markers in the serum and aqueous humor of glaucoma patients did not differ significantly between males and females (S1, S2 and S3 Tables).

**Table 1:** Meta-regression for oxidative markers in serum.

Covariates	Coefficient (95%CI)	p-value
**Population**		
Sex (Male as reference)	**0.94 (0.59, 1.28)**	**< .001**
Age	**-1.19 (-1.54, 0.84)**	**< .001**
**Oxidative stress markers**		
Total oxidative stress vs other	^‡^	^‡^
Malonyldialdehyde vs other	**1.46 (0.16, 2.77)**	**.03**
Protein carbonyl vs other	^‡^	^‡^
8 OhdG vs other	^‡^	^‡^
**Type of glaucoma**^†^		
POAG vs PACG	19.2 (-27.3, 65.7)	.36
POAG vs PEG	20.2 (-21.5, 62.0)	.29
PACG vs PEG	1.04 (-28.1, 26.0)	.93
Difference POAG/controls and PACG/controls	**12.7 (8.78, 16.6)**	**< .001**
Difference POAG/controls and PEG/controls	-0.42 (-2.86, 2.00)	0.69
Difference PEG/controls and PACG/controls	**12.2 (8.96, 15.5)**	**< .001**

95%CI: 95% confidence intervals; PACG: primary angle closure glaucoma; PEG: pseudoexfoliation glaucoma; POAG: primary open angle glaucoma.

^†^: Separate models were used to assess all combinations. As coefficient (95%CI) and p-value of other covariates were identical regarding all models, we report all the combinations in the same table in order to avoid duplications.

^‡^: Dropped because of collinearity.

#### Age

Levels of oxidative stress markers in the serum of glaucoma patients decreased with age (effect size = –1.19; 95%CI –1.54 to 0.84, P < 0.001) (Table 1). There was no age effect on the levels of oxidative stress markers in aqueous humor and antioxidative stress markers in the serum and aqueous humor of glaucoma patients (S1, S2 and S3 Tables)

#### Markers of oxidative stress

Malonyldialdehyde was higher than other of oxidative stress markers in the serum of glaucoma patients (effect size = 1.46; 95%CI 0.16–2.77, P = 0.03) (Table 1). Insufficient data precluded further analyses for levels of oxidative stress markers in aqueous humor in glaucoma patients (S1 Table)

#### Markers of antioxidative stress

No single antioxidative stress marker demonstrated a greater difference than any other marker in glaucoma patients (S2 and S3 Tables)

#### Type of glaucoma

In patients with glaucoma, effect sizes for oxidative and antioxidative stress markers did not differ significantly between the types of glaucoma (POAG, PACG, and PEG). The differences in serum levels of oxidative stress markers between glaucoma patients and controls were significantly higher in POAG vs PACG (effect size = 12.7; 95%CI 8.78–16.6, P < 0.001), and higher in PEG vs PACG (effect size = 12.2; 95%CI 8.96–15.5, P < 0.001) (Table 1). There were no differences in the levels of oxidative stress markers in aqueous humor and antioxidative stress markers in the serum and aqueous humor either between patients with different types of glaucoma or between glaucoma patients and controls (S1, S2 and S3 Tables).

## Discussion

The major findings were an overall increase of oxidative stress markers in glaucoma (effect size = 1.64; 95%CI 1.20–2.09), ranging from an effect size of 1.29 in serum (95%CI 0.84–1.74) to 2.62 in aqueous humor (95%CI 1.60–3.65). Malonyldialdehyde seemed the best serum biomarker of oxidative stress (effect size = 1.46 vs other biomarkers; 95%CI 0.16–2.77). Despite a decrease in serum antioxidative stress markers (effect size = –0.41; 95%CI –0.72 to –0.11), the level of some aqueous humor antioxidative stress markers increased (superoxide dismutase, effect size = 3.53; 95%CI 1.20–5.85 and glutathione peroxidase, effect size = 6.60; 95%CI 3.88–9.31), which could be a protective response of the eye against oxidative stress.

### Oxidative stress and glaucoma

In our study, we demonstrated an increase in oxidative stress in glaucoma in line with the literature [14,50]. With malonyldialdehyde seeming to be a good marker to evaluate oxidative stress in serum and aqueous humor. Malonyldialdehyde is a product of reactive oxygen species acting on polyunsaturated fat [51]. Evaluation of malonyldialdehyde levels remains a useful indicator of lipid peroxidation linked with oxidative stress [52]. All studies compared the aqueous humor of glaucoma patients with patients undergoing cataract surgery. Though it has been suggested that cataracts enhance oxidative stress [53,54], our meta-analyses demonstrated significant that the most important effect size for oxidative stress was in aqueous humor. A perturbation of the oxidant/antioxidant balance in the aqueous humor causes an increase in the production of reactive oxygen species which may lead to trabecular meshwork damage [17,55] in predisposed patients [56]. The trabecular meshwork regulates the outflow of aqueous humor via the anterior chamber [57]. However, this structure has the greatest sensitivity to the consequences of oxidative stress [14]. Intraocular pressure rises and leads to optic nerve and retinal ganglion cell degeneration, which are already weakened by chronic oxidative stress [58].

### Antioxidative defense system in glaucoma

We demonstrated a decreased total antioxidant status in serum and aqueous humor in glaucoma. However, the levels of two common antioxidative stress markers, i.e. superoxide dismutase and glutathione peroxidase, increased in aqueous humor. This could be a protective response of the eye against oxidative stress [36,59], and may decrease in the long term [37,55]. More interestingly, the trabecular meshwork is a metabolically active tissue containing key enzymes involved in protecting against oxidative stress, in particular the two important enzymes superoxide dismutase and glutathione peroxidase [60]. These two enzymes play a major role by removing the excess H_2_O_2_. Despite its toxicity when it exceeds the physiological values [61], the aqueous humor normally contains H_2_O_2_, which is an essential component of several signal-transduction pathways [62]. The removal of H_2_O_2_ uses glutathione as a cofactor and decreases glutathione levels [47]. As glutathione is involved in ascorbic acid metabolism, its depletion produces ascorbyl radicals that cannot be regenerated to ascorbic acid [47]. This might explain the decreased vitamin C levels in serum and aqueous humor found in our meta-analyses. No significant changes were found in the levels of catalase, which could be explain by a change in its absorbance spectrum following its combination with nitric oxide [63]. It has therefore been postulated that some chronic damage in the trabecular meshwork decreases the levels of superoxide dismutase and glutathione peroxidase and leads to increased oxidative stress in the anterior chamber [55].

### Type of glaucoma and clinical application

Among the few studies comparing the levels of oxidative or antioxidative stress markers between the different types of glaucoma [35,36,38,40,42], only one study reported a significant difference in malonyldialdehyde serum levels between POAG and PEG [35]. We demonstrated a higher level of oxidative stress markers in patients’ serum compared to controls in POAG vs PACG (effect size = 12.7; 95%CI 8.78–16.6), and in PEG vs PACG (effect size = 12.2; 95%CI 8.96–15.5). Even if glaucoma is a multifactorial disease [7,10], systemic oxidative stress may play an important role in the pathophysiology of POAG and PEG. Despite growing research on antioxidant therapy to prevent glaucoma, in both in vitro [64] and in vivo studies with animals [65] and humans [66], antioxidative supplementation has shown contradictory [66,67,68]. However, there were promising results of supplementation in endothelial function [69], some specific clinical conditions [70] and overall mortality [71]. Therefore, as oxidative stress also contributes to the pathogenesis of systemic clinical conditions [72] and other ocular conditions [73], antioxidative supplementation may not be neglected. Although most hypotheses of aging are related to an increase in oxidative stress [74,75,76], we found that levels of oxidative stress decreased with aging in glaucoma. Moreover, we found higher levels of oxidative stress markers in men with glaucoma, compared with women. Even if no study reported this relationship in glaucoma, this finding is in agreement with higher systemic levels of antioxidative stress in men in the general population [77,78]. The higher prevalence of glaucoma in men may be linked to increased oxidative stress. These relationships should be further investigated, as well as the putative benefits to measure malonyldialdehyde levels in serum in clinical practice.

### Limitations

Our study has some limitations. All the studies included were cross-sectional. However, we demonstrated the putative role of oxidative stress in glaucoma. Though there were similarities between the inclusion criteria, they were not identical. In particular for glaucoma patients, some studies did not clearly defined the definition used for glaucoma, and exclusion criteria also differed between studies which may have affected our results. In addition, some controls could have another cause of increased oxidative stress, such as cataract [30,33,36,37,38,41,42,44,47]. This may have minimized the differences we reported in oxidative stress levels between glaucoma participants and controls. Limiting our meta-analysis to studies sharing the same inclusion and exclusion criteria was not feasible due to the limited data. Moreover, all studies were monocentric, limiting the generalizability of our results. Many markers were reported in only one study precluding further comparisons between markers of oxidative or antioxidative stress levels. However, we demonstrated the potential use of the measure of malonyldialdehyde. Even if several studies assessed the levels of oxidative or antioxidative stress within the aqueous humor, it is not necessarily easily applicable in daily clinical practice. For ethical reasons and feasibility, there is no study evaluating in vivo oxidative markers in vitreous, retina and optic nerve. However, our meta-analyses demonstrated the potential for assessing serum oxidative stress levels. Unfortunately, the lack of details surrounding the disease, such as the beginning of glaucoma or the treatment after diagnosis, precluded further analyses.

## Conclusion

We demonstrated an overall increase of oxidative stress markers in glaucoma (effect size = 1.64; 95%CI 1.20–2.09), ranging from an effect size of 1.29 in serum (95%CI 0.84–1.74) to 2.62 in aqueous humor (95%CI 1.60–3.65). Malonyldialdehyde seemed the best biomarkers of oxidative stress in serum. Despite a decrease of antioxidative stress markers in serum, some increased in the aqueous humor which could be a protective response of the eye against oxidative stress. Implications of oxidative stress in the pathophysiology of glaucoma and the potentially interesting therapeutic approach should be studied further.

## Supporting Information

S1 Appendix PRISMA Checklist. (DOCX)

S1 Fig Meta-analysis of oxidative stress markers from serum and aqueous humor in glaucoma. 95%CI: 95% confidence intervals; -: Unknown; PACG: primary angle closure glaucoma; PEG: pseudoexfoliation glaucoma; POAG: primary open angle glaucoma; 8 OhdG: 8-hydroxydeoxyguanosin; AOPP: Advanced oxidation protein product; IMA: Ischemied modified albumin.
(TIF)

S2 Fig Funnel plots of oxidative stress markers from serum and aqueous humor in glaucoma. (TIF)

S3 Fig Meta-analysis of oxidative stress markers from serum in glaucoma after exclusion of studies not evenly distributed around the base of the funnel. 95%CI: 95% confidence intervals; -: Unknown; PACG: primary angle closure glaucoma; PEG: pseudoexfoliation glaucoma; POAG: primary open angle glaucoma; 4NHE: 4-hydroxynonenal; 8 OhdG: 8-hydroxydeoxyguanosin; AOPP: Advanced oxidation protein product; IMA: Ischemied modified albumin.
(TIF)

S4 Fig Meta-analysis of antioxidative stress markers from serum and aqueous humor in glaucoma. 95%CI: 95% confidence intervals; -: Unknown; PACG: primary angle closure glaucoma; PEG: pseudoexfoliation glaucoma; POAG: primary open angle glaucoma; GST: Glutathione S transferase.
(TIF)

S5 Fig Funnel plots of antioxidative stress markers from serum and aqueous humor in glaucoma. (TIF)

S6 Fig Meta-analysis of antioxidative stress markers from serum in glaucoma after exclusion of studies not evenly distributed around the base of the funnel. 95%CI: 95% confidence intervals; -: Unknown; PACG: primary angle closure glaucoma; PEG: pseudoexfoliation glaucoma; POAG: primary open angle glaucoma; GPx: Glutathione peroxidase; SOD: Superoxide dismutase; TAS: Total antioxidant status.
(TIF)

S7 Fig Meta-analysis of antioxidative stress markers from aqueous in glaucoma after exclusion of studies not evenly distributed around the base of the funnel. 95%CI: 95% confidence intervals; -: Unknown; PACG: primary angle closure glaucoma; PEG: pseudoexfoliation glaucoma; POAG: primary open angle glaucoma; GPx: Glutathione peroxidase; GST: Glutathione S transferase; SOD: Superoxide dismutase; TAS: Total antioxidant status.
(TIF)

S1 Table Meta-regression for oxidative markers in aqueous humor. 95%CI: 95% confidence intervals; PACG: primary angle closure glaucoma; PEG: pseudoexfoliation glaucoma; POAG: primary open angle glaucoma.
(DOCX)

S2 Table Meta-regression for antioxidative markers in serum. 95%CI: 95% confidence intervals; PACG: primary angle closure glaucoma; PEG: pseudoexfoliation glaucoma; POAG: primary open angle glaucoma.
(DOCX)

S3 Table Meta-regression for antioxidative markers in aqueous humor. 95%CI: 95% confidence intervals; PACG: primary angle closure glaucoma; PEG: pseudoexfoliation glaucoma; POAG: primary open angle glaucoma.
(DOCX)
