# Supplementary figures and images for: Oxidative and Anti-Oxidative Stress Markers in Chronic Glaucoma: A Systematic Review and Meta-Analysis
Source: PLoS One. 2016 Dec 1;11(12):e0166915. doi: 10.1371/journal.pone.0166915 (PMC5131953; doi:10.1371/journal.pone.0166915)

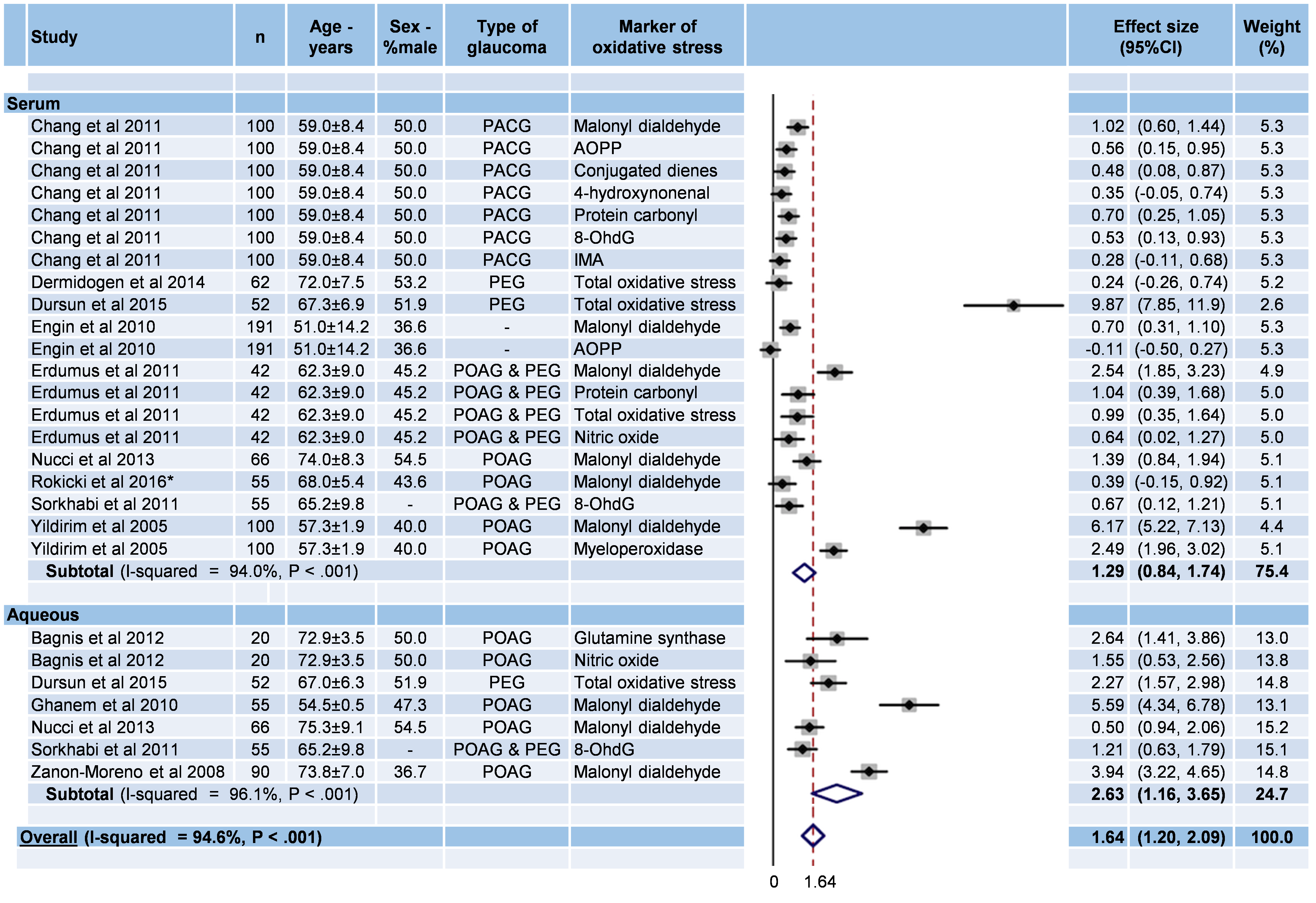

Supplement: S1 Fig — 95%CI: 95% confidence intervals; -: Unknown; PACG: primary angle closure glaucoma; PEG: pseudoexfoliation glaucoma; POAG: primary open angle glaucoma; 8 OhdG: 8-hydroxydeoxyguanosin; AOPP: Advanced oxidation protein product; IMA: Ischemied modified albumin. (TIF) [file pone.0166915.s002.tif]

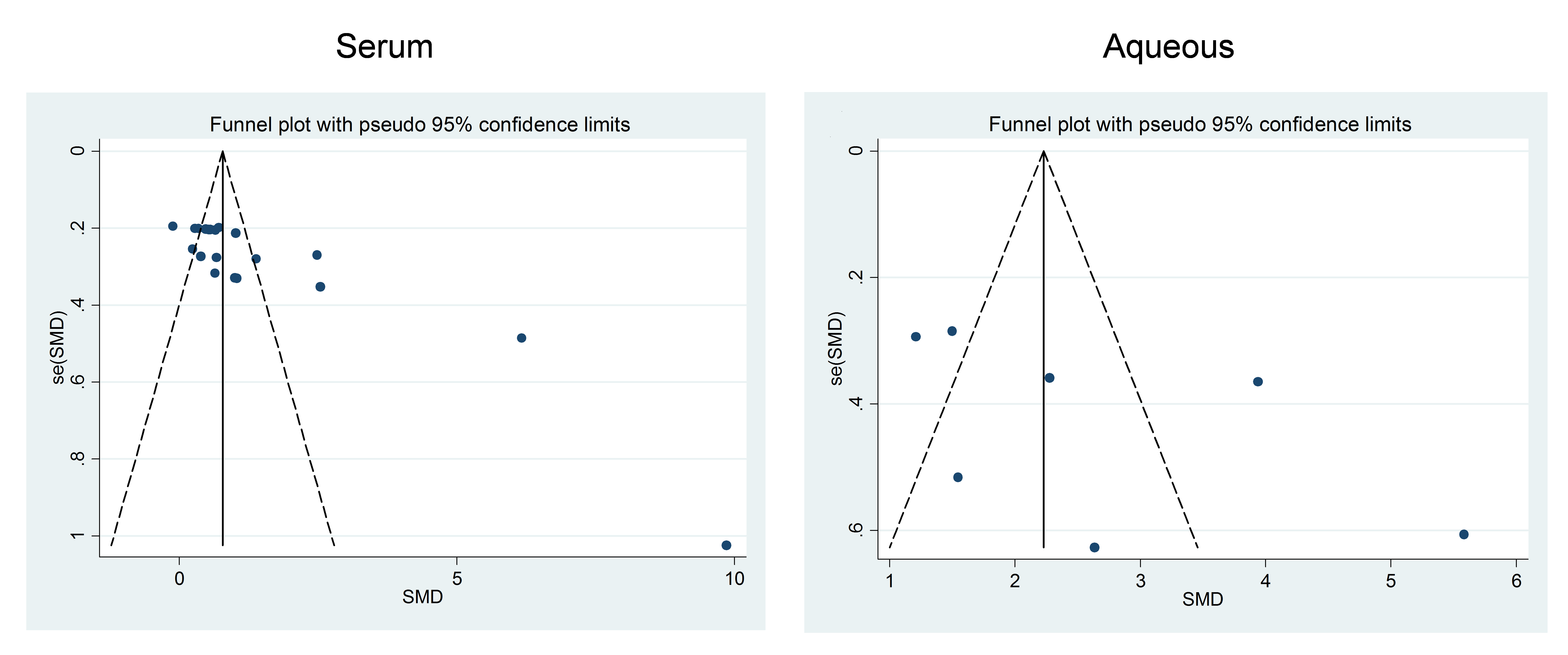

Supplement: S2 Fig — (TIF) [file pone.0166915.s003.tif]

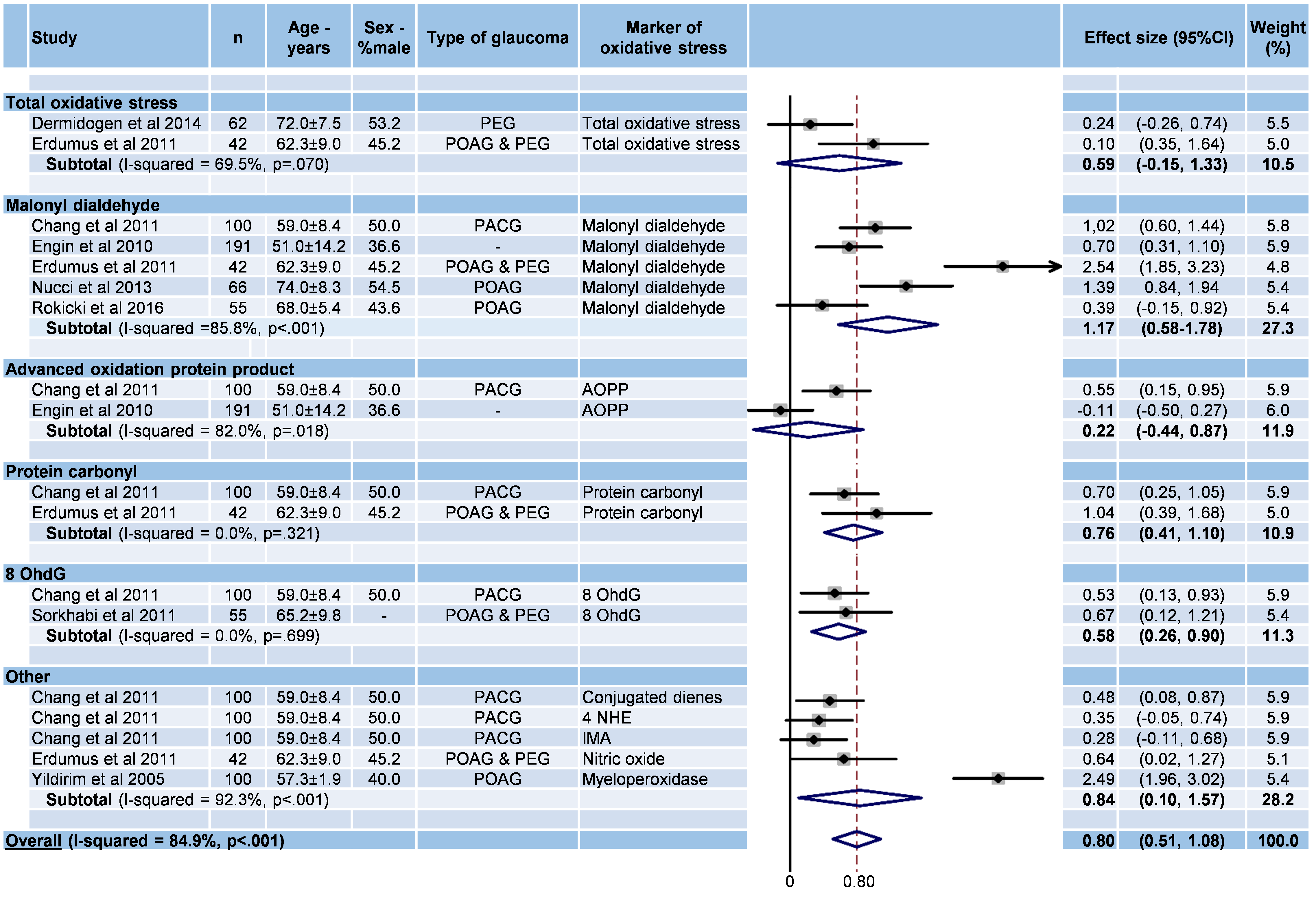

Supplement: S3 Fig — 95%CI: 95% confidence intervals; -: Unknown; PACG: primary angle closure glaucoma; PEG: pseudoexfoliation glaucoma; POAG: primary open angle glaucoma; 4NHE: 4-hydroxynonenal; 8 OhdG: 8-hydroxydeoxyguanosin; AOPP: Advanced oxidation protein product; IMA: Ischemied modified albumin. (TIF) [file pone.0166915.s004.tif]

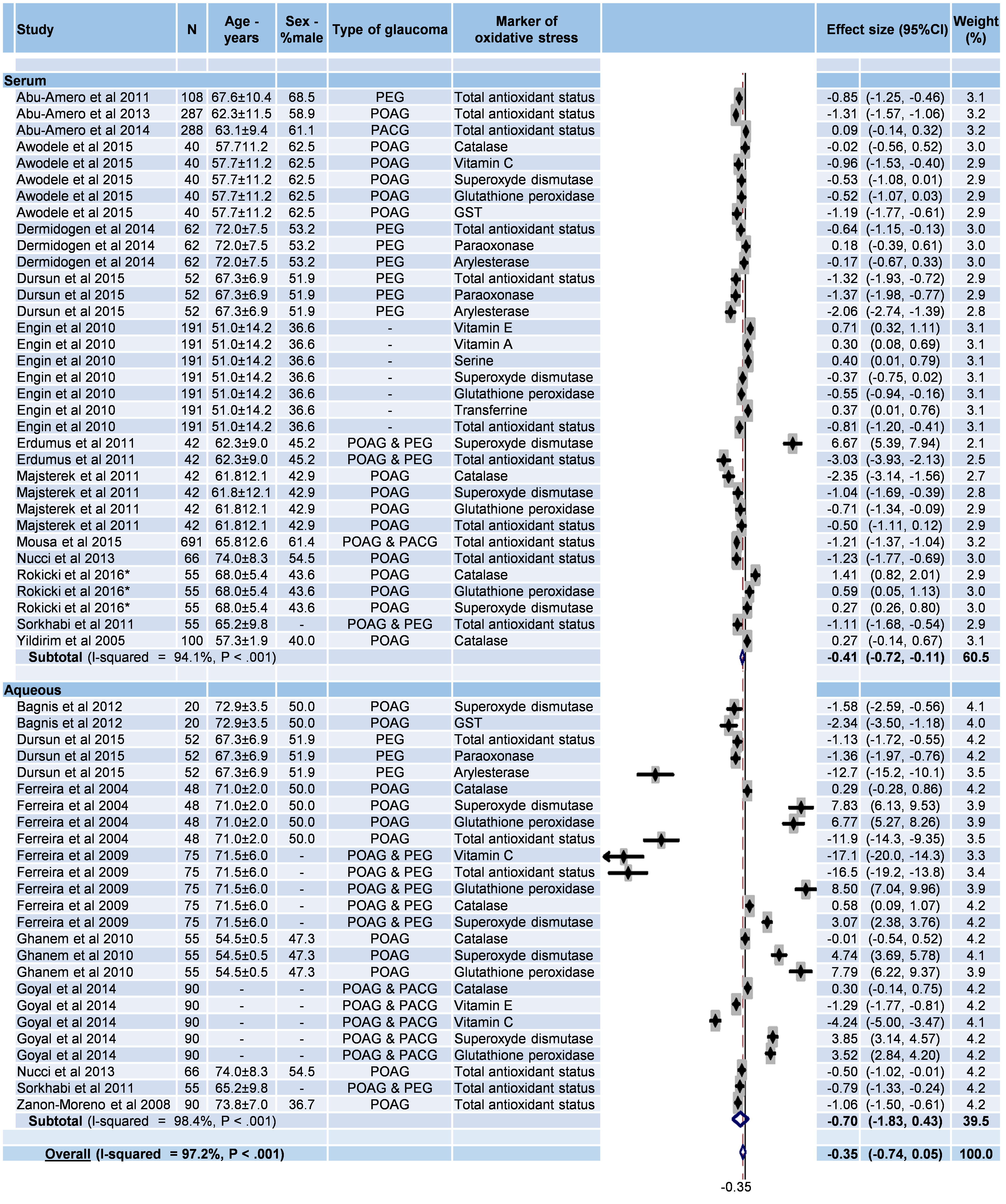

Supplement: S4 Fig — 95%CI: 95% confidence intervals; -: Unknown; PACG: primary angle closure glaucoma; PEG: pseudoexfoliation glaucoma; POAG: primary open angle glaucoma; GST: Glutathione S transferase. (TIF) [file pone.0166915.s005.tif]

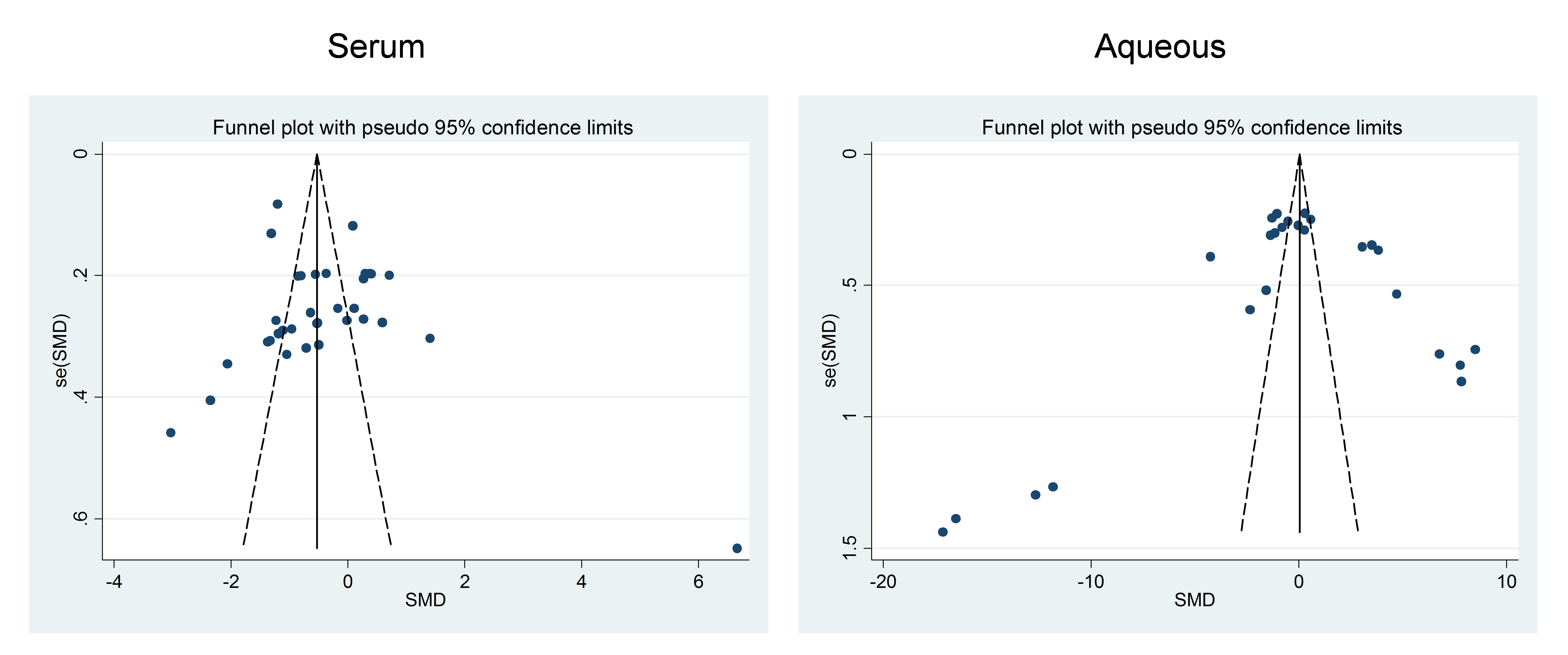

Supplement: S5 Fig — (TIF) [file pone.0166915.s006.tif]

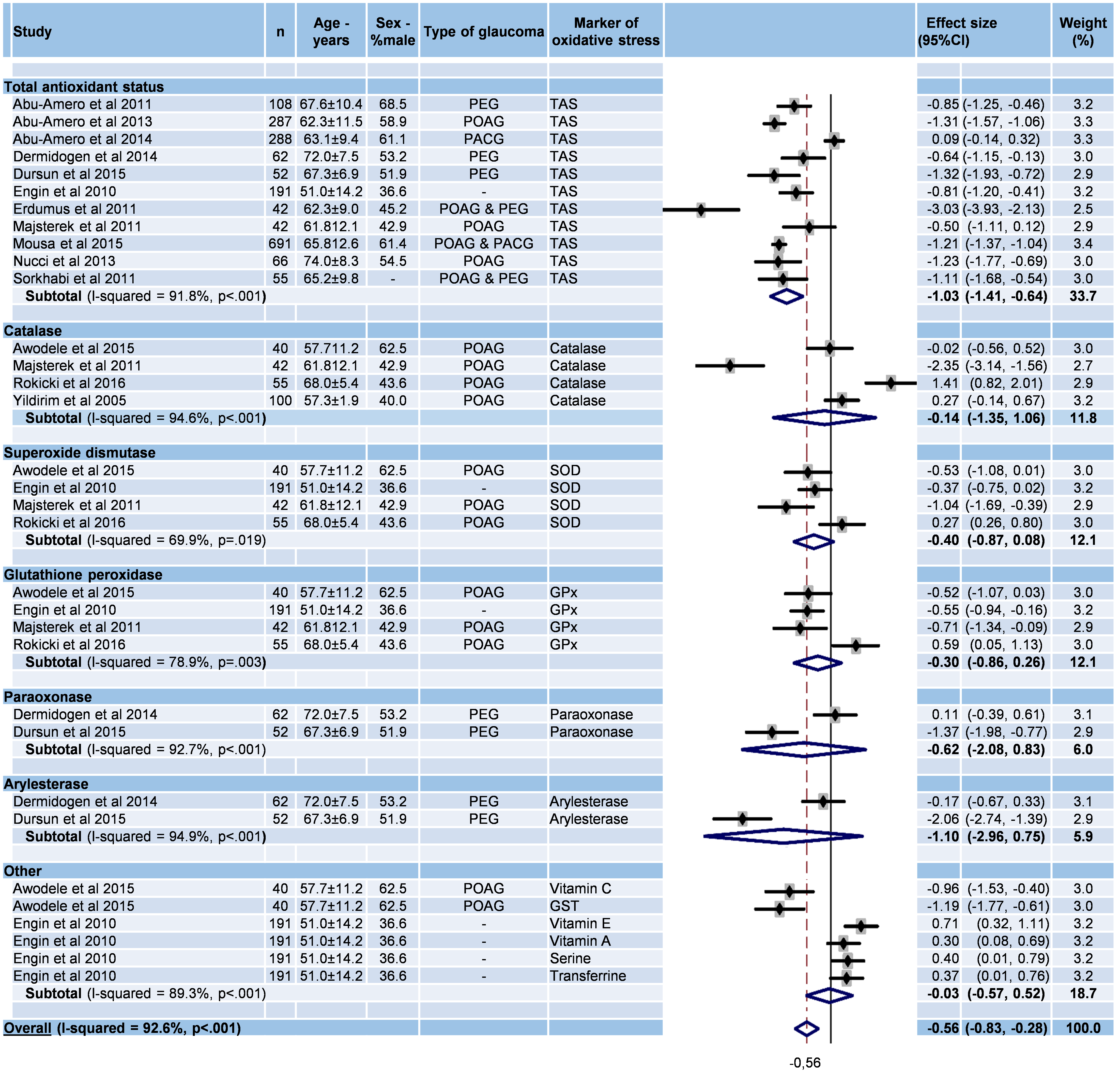

Supplement: S6 Fig — 95%CI: 95% confidence intervals; -: Unknown; PACG: primary angle closure glaucoma; PEG: pseudoexfoliation glaucoma; POAG: primary open angle glaucoma; GPx: Glutathione peroxidase; SOD: Superoxide dismutase; TAS: Total antioxidant status. (TIF) [file pone.0166915.s007.tif]

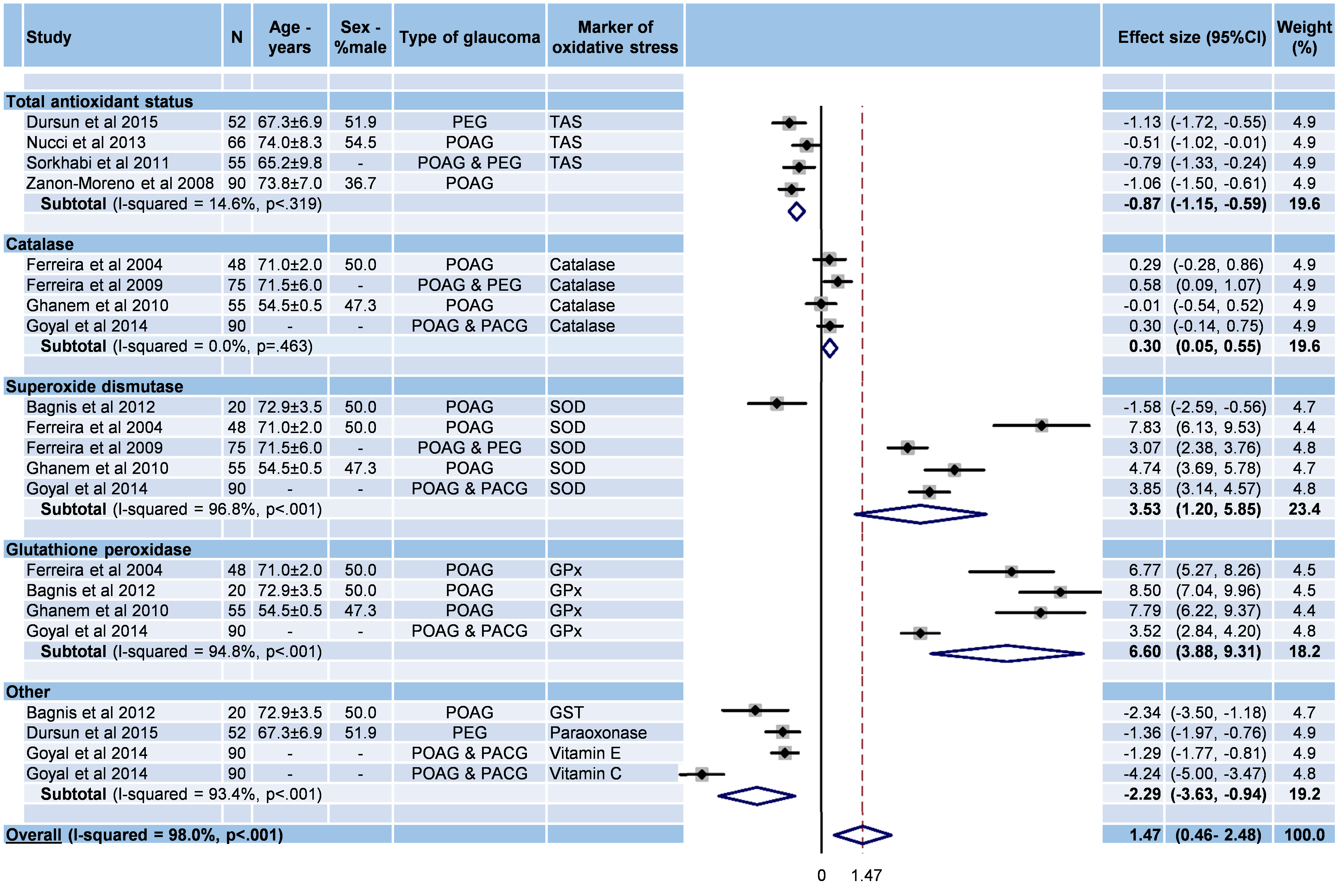

Supplement: S7 Fig — 95%CI: 95% confidence intervals; -: Unknown; PACG: primary angle closure glaucoma; PEG: pseudoexfoliation glaucoma; POAG: primary open angle glaucoma; GPx: Glutathione peroxidase; GST: Glutathione S transferase; SOD: Superoxide dismutase; TAS: Total antioxidant status. (TIF) [file pone.0166915.s008.tif]
