# Supplementary material for: Oxidative and Anti-Oxidative Stress Markers in Chronic Glaucoma: A Systematic Review and Meta-Analysis
Source: PLoS One. 2016 Dec 1;11(12):e0166915. doi: 10.1371/journal.pone.0166915 (PMC5131953; doi:10.1371/journal.pone.0166915)
Supplement: S1 Table — 95%CI: 95% confidence intervals; PACG: primary angle closure glaucoma; PEG: pseudoexfoliation glaucoma; POAG: primary open angle glaucoma. (DOCX) [file pone.0166915.s009.docx]

**S1 Table.** Meta-regression for oxidative markers in aqueous humor.

95%CI: 95% confidence intervals; PACG: primary angle closure glaucoma; PEG: pseudoexfoliation glaucoma; POAG: primary open angle glaucoma.

|  |  |  |
| --- | --- | --- |
| **Covariates** | **Coefficient (95%CI)** | **p-value** |
| **Population** |  |  |
| Sex (Male as reference) | -0.07 (-0.64, 0.49) | 0.64 |
| Age | 0.04 (-0.20, 0.28) | 0.56 |
| **Oxidative stress markers** |  |  |
| Malonyl dialedhyde vs other | ^‡^ | ^‡^ |
| **Type of glaucoma**^†^ |  |  |
| POAG vs PACG | Insufficient data | - |
| POAG vs PEG | 2.73 (-4.02, 9.49) | 0.22 |
| PACG vs PEG | Insufficient data | - |
| Difference POAG/controls and PACG/controls | Insufficient data | - |
| Difference POAG/controls and PEG/controls | 0.53 (-3.29, 4.36) | 0.61 |
| Difference PACG/controls and PEG/controls | Insufficient data | - |
|  |  |  |

^†^: Separate models were used to assess all combinations. As coefficient (95%CI) and p-value of other covariates were identical regarding all models, we report all the combinations in the same table in order to avoid duplications.

^‡^: Dropped because of collinearity.
