# Supplementary material for: Oxidative and Anti-Oxidative Stress Markers in Chronic Glaucoma: A Systematic Review and Meta-Analysis
Source: PLoS One. 2016 Dec 1;11(12):e0166915. doi: 10.1371/journal.pone.0166915 (PMC5131953; doi:10.1371/journal.pone.0166915)
Supplement: S2 Table — 95%CI: 95% confidence intervals; PACG: primary angle closure glaucoma; PEG: pseudoexfoliation glaucoma; POAG: primary open angle glaucoma. (DOCX) [file pone.0166915.s010.docx]

**S2 Table.** Meta-regression for antioxidative markers in serum.

95%CI: 95% confidence intervals; PACG: primary angle closure glaucoma; PEG: pseudoexfoliation glaucoma; POAG: primary open angle glaucoma.

|  |  |  |
| --- | --- | --- |
| **Covariates** | **Coefficient (95%CI)** | **p-value** |
| **Population** |  |  |
| Sex (Male as reference) | -0.05 (-0.16, 0.6) | .35 |
| Age | 0.08 (-0.04, 0.21) | .18 |
| **Antioxidative stress markers**^†^ |  |  |
| Total antioxidant status vs other | -0.29 (-1.53, 0.94) | .62 |
| Catalase vs other | 0.57 (-0.85, 1.98) | .40 |
| Superoxide dismutase vs other | -1.15 (-1.76, 1.45) | .84 |
| Glutathione peroxidase vs other | 0.25 (-1.35, 1.85) | .74 |
| Paraoxonase vs other | 0.16 (-1.55, 1.88) | .84 |
| Arylesterase vs other | 0.51 (-2.21, 1.18) | .53 |
| **Type of glaucoma**^†^ |  |  |
| POAG vs PACG | 6.84 (-606, 619) | .98 |
| POAG vs PEG | -6.94 (-407, 393) | .97 |
| PACG vs PEG | -13.8 (-584, 556) | .96 |
| Difference POAG/controls and PACG/controls | 1.54 (-0.95, 4.04) | .21 |
| Difference POAG/controls and PEG/controls | -0.30 (-1.83, 1.23) | .68 |
| Difference PACG/controls and PEG/controls | -1.84 (-0.47, 4.15) | .11 |
|  |  |  |

^†^: Separate models were used to assess all combinations. As coefficient (95%CI) and p-value of other covariates were identical regarding all models, we report all the combinations in the same table in order to avoid duplications.
