# Supplementary material for: Oxidative and Anti-Oxidative Stress Markers in Chronic Glaucoma: A Systematic Review and Meta-Analysis
Source: PLoS One. 2016 Dec 1;11(12):e0166915. doi: 10.1371/journal.pone.0166915 (PMC5131953; doi:10.1371/journal.pone.0166915)
Supplement: S3 Table — 95%CI: 95% confidence intervals; PACG: primary angle closure glaucoma; PEG: pseudoexfoliation glaucoma; POAG: primary open angle glaucoma. (DOCX) [file pone.0166915.s011.docx]

**S3 Table.** Meta-regression for antioxidative markers in aqueous humor.

95%CI: 95% confidence intervals; PACG: primary angle closure glaucoma; PEG: pseudoexfoliation glaucoma; POAG: primary open angle glaucoma.

|  |  |  |
| --- | --- | --- |
| **Covariates** | **Coefficient (95%CI)** | **p-value** |
| **Population** |  |  |
| Sex (Male as reference) | 0.25 (-2.02, 2.52) | .81 |
| Age | -.025 (-0.95, 0.46) | .45 |
| **Antioxidative stress markers**^†^ |  |  |
| Total antioxidant status vs other | -3.35 (-11.9, 5.19) | .40 |
| Catalase vs other | -2.71 (-14.1, 8.70) | .61 |
| Superoxide dismutase vs other | 3.54 (-5.80, 12.9) | .41 |
| Glutathione peroxidase vs other | 6.74 (-3.75, 17.2) | .18 |
| Paraoxonase vs other | Insufficient data | - |
| Arylesterase vs other | Insufficient data | - |
| **Type of glaucoma**^†^ |  |  |
| POAG vs PACG | No data | - |
| POAG vs PEG | 630 (-1301, 2561) | .48 |
| PACG vs PEG | No data | - |
| Difference POAG/controls and PACG/controls | No data | - |
| Difference POAG/controls and PEG/controls | -7.84 (-29.2, 13.5) | .43 |
| Difference PACG/controls and PEG/controls | No data | - |
|  |  |  |

^†^: Separate models were used to assess all combinations. As coefficient (95%CI) and p-value of other covariates were identical regarding all models, we report all the combinations in the same table in order to avoid duplications.
